# Larval-Transcriptome Dynamics of *Ectropis grisescens* Reveals Differences in Virulence Mechanism between Two EcobNPV Strains

**DOI:** 10.3390/insects13121088

**Published:** 2022-11-26

**Authors:** Xinxin Zhang, Yang Mei, Hong Li, Meijun Tang, Kang He, Qiang Xiao

**Affiliations:** 1 Ministry of Agriculture Key Laboratory of Tea Quality and Safety Control, Tea Research Institute, Chinese Academy of Agricultural Sciences, Hangzhou 310008, China; 2 Key Laboratory of Biology of Crop Pathogens and Insects of Zhejiang Province, Institute of Insect Sciences, College of Agriculture and Biotechnology, Zhejiang University, Hangzhou 310058, China

**Keywords:** *Ectropis grisescens*, *Ectropis obliqua* nucleopolyhedrovirus, virulence difference, transcriptome

## Abstract

**Simple Summary:**

Baculoviruses are virulent pathogens of a number of important insect-pest species. *Ectropis grisescens* is a serious leaf-eating pest in tea plantations, which causes incalculable losses to tea production. *Ectropis obliqua* nucleopolyhedrovirus (EcobNPV), as a biological insecticide, has been applied to control *E. grisescens*. Most prior detailed studies of EcobNPV-strain infections have focused on virulence bioassay. In the previous period, our group screened a highly efficient strain, EcobNPV-QF4, which is more virulent for *E. grisescens* than EcobNPV-QV. In this study, we compared the virulence of the strains using the leaf-disc method and generated larval-transcriptomes of infected *E. grisescens* at different time-points after infection. Understanding the transcriptional responses of the host to viral infection is critically important for understanding the mechanism of the differences in virulence between the two strains.

**Abstract:**

The biological insecticide, *Ectropis obliqua* nucleopolyhedrovirus (EcobNPV), has been applied to control the major tea-pest *Ectropis grisescens*. Previously, the virus strain EcobNPV-QF4 showed higher a mortality rate (58.2% vs. 88.2%) and shorter median lethal-time (13.9 d vs. 15.4 d) on *E. grisescens* than the strain EcobNPV-QV. However, the mechanism of the difference in virulence between the two strains remains unclear. Using the leaf-disc method, we detected the virulence of the two strains on 3^rd^-instar larvae, and found that median lethal-dose (LD_50_) of EcobNPV-QF4 is 55-fold higher than that of EcobNPV-QV (4.35 × 10^8^ vs. 7.89 × 10^6^). Furthermore, fourteen larva transcriptomes of *E. grisescens* were subsequently sequenced at seven time-points after ingestion of the two virus strains, yielding 410.72 Gb of raw reads. Differential gene-expression analysis shows that 595, 87, 27, 108, 0, 12, and 290 genes were up-regulated in EcobNPV-QF4 at 0, 2, 6, 12, 24, 36 h and 48 h post ingestion (hpi), while 744, 68, 152, 8, 1, 0, 225 were down-regulated. KEGG enrichment showed that when the virus first invades (eats the leaf-discs), EcobNPV-QF4 mainly affects pathways such as ribosome (*p*-value = 2.47 × 10^−29^), and at 48 hpi EcobNPV-QF4, causes dramatic changes in the amino-acid-synthesis pathway and ribosome pathway (*p*-value = 6.94 × 10^−13^) in *E. grisescens*. Among these, thirteen key genes related to immunity were screened. The present study provides the first ever comprehensive analysis of transcriptional changes in *E. grisescens* after ingestion of the two strains of EcobNPV.

## 1. Introduction

Tea is a popular functional-food worldwide, and China is the largest tea planting and producing country [1]. *Ectropis obliqua* and *Ectropis grisescens* (Lepidoptera, Geometridae, Ennominae) are the most destructive primary defoliators in tea plantations in China [2]. Due to the similar morphology, tea growers took them as the same species for a long time. The reproductive capacity, virus susceptibility and geographical distribution of the “tea looper” were then compared with the morphological and molecular characterization in different regions of China, revealing that they are totally different species [3]. Compared with *E. obliqua*, *E. grisescens* have a larger body, faster development-rate, greater reproductive-capacity and broader habitat-distribution [4,5,6].

As a member of Baculoviridae, *Ectropis obliqua* nucleopolyhedrovirus (*Ecob*NPV) is pathogenic for invertebrates, particularly members of Insecta, such as the orders of Lepidoptera, Hymenoptera, and Diptera [7]. EcobNPV has been used as a species-specific bio-control agent for *Ectropis* spp. [8]. However, for the more widely distributed *E. grisescens*, the virus shows significant discrepancy in susceptibility and virulence among different strains. The EcobNPV-QF4 strain shows a higher mortality of 88.2% for *E. grisescens* on the 12th day post-infection, compared with 58.2% for the EcobNPV-QV strain, and the median lethal-time (LT_50_) of EcobNPV-QF4 on *E. grisescens* was 13.9 d, compared with 15.4 d for EcobNPV-QV [9]. The EcobNPV-QF4 genome contains 128 genes and three homologous regions, and the EcobNPV-QV genome has 127 genes and three homologous regions [10].

The baculovirus generally produces two types of viral particles during the infection process: occlusion-derived virus (ODVs) and budded virus (BVs) [11]. When the larvae first ingest occlusion bodies (OBs), the virus enters the midgut and dissolves in the alkaline environment, releasing ODVs. The ODVs then bind specifically to the apical microvilli on the surface of the midgut epithelial-cells across the periplasm, releasing viral nucleocapsids into the host cytoplasm [12]. Viral genome is then released into the host-cell nucleus through the nuclear pore, and host genes and cytoplasmic matrix are recruited to initiate the viral transcription, replication, assembly, and production of the nucleocapsid [13]. Virus particles are wrapped by fusion proteins that accumulate near the cell membrane when they cross the host-cell membrane to form BVs. The BVs reach other tissues of the insect through the trachea and hemolymph, eventually resulting in systemic infection, which causes debilitation and often the death of the host [14]. However, the mechanism of the EcobNPV infection of *E. grisescens* has not been thoroughly studied, and whether a similar repertoire of genes in *E. grisescens* were induced in response to the infection of different strains of EcobNPV, is still unclear.

High-throughput transcriptome sequencing of BmNPV-infected brains of the silkworm *Bombyx mori* revealed differentially expressed genes (DEGs) involved in synaptic transmission, circadian rhythms, and the serotonin-receptor signaling pathway at 96 hpi, which were apparently related to effects on locomotor activity [15]. This suggested that oxidative stress might result from the activation of silkworm defensive-functions, following BmNPV illness. In addition, multiple metabolic activities such as oxidative phosphorylation were affected, which have biological relevance to the antiviral mechanism of silkworm [16].

To gain insight into the immune response of *E. grisescens* ingestion of the two EcobNPV strains, the virulence was first determined using a leaf-disc bioassay. Larvae transcriptomes were sampled and analyzed at 0, 2, 6, 12, 24, 36, and 48 hpi, and DEGs were identified between strains at each time-point. KEGG enrichment analysis was carried out to investigate the DEGs-involved pathways accounting for the discrepancy of virulence. These results will provide a useful reference for the study of the mechanism underlying the differences in virulence of the two strains.

## 2. Materials and Methods

### 2.1. Viruses and Insects

*E. grisescens* young larvae were collected from tea plantations in Yueqing City (120.98° E, 28.11° N), Wenzhou City, Zhejiang Province, China. The larvae were reared on fresh tea-leaves for at least 3 generations under laboratory conditions, at 25 ± 1 °C with 70–80% relative humidity and a photoperiod of 12:12 (light/dark). Both EcobNPV-QV and EcobNPV-QF4 stains were obtained by in vivo-propagation technology [9,17] and stored at 4 °C, for use.

### 2.2. Virulence Bioassay

The virulence of the two strains for *E. grisescens* was measured using the 3rd-instar larvae with the leaf-disc method [5]. Fresh tea-leaf discs of 9-mm were made with a circular punch, and 10 µL of virus solution was added to the leaf disc for eating. Virus solutions were diluted with distilled water at the following concentrations: 2 × 10^6^, 2 × 10^7^, 2 × 10^8^, 2 × 10^9^ OB (occlusion body)/mL. After starvation of 6 h, the larvae were individually placed into a 24-well plate with fresh tea-leaf discs and reared for 8 h, then transferred to clean and non-toxic jars with fresh tea-leaves for feeding, until pupation. Twenty insects were tested per dose with four replicates. Mortality was recorded daily, until all the larvae either pupated or died. Adjusted mortality was calculated with distilled-water treatment as control.

### 2.3. Preparation of the Infected E. grisescens Larvae

First, the fresh leaf-discs of the 9-mm plates were prepared with 10 µL of virus solution (2 × 10^8^ OB/mL). The 3rd-instar larvae infected with each strain were in starvation for 6 h before infection. Next, the leaf-disc was placed on a petri dish with wet filter-paper on the bottom. After the liquid was dried, all larvae were individually fed a 24-well chamber plate, to make sure that each larva ate up the entire leaf-disc.The virus-free tea leaves were then added into the glass bottle, for regular feeding.

### 2.4. Library Construction and Sequencing

To obtain consistent transcriptional data, whole-body homogenates of *E. grisescens* larvae at 0, 2, 6, 12, 24, 36 and 48 h post ingestion (hpi) were prepared for RNA sequencing. Three insects were tested in each assay, with three replicates. The 0 hpi time-point corresponds to the point at which the entire leaf disc was consumed. Total RNA was extracted using TRI REAGENT (Molecular Research Center, Cincinnati, OH, USA). Library construction and sequencing were performed using the Beijing Novogene Technology Co., LTD. (Beijing, China). RNA integrity was assessed using the RNA Nano 6000 Assay Kit of the Bioanalyzer 2100 system (Agilent Technologies, Santa Clara, CA, USA), using total RNA as input material. Briefly, RNA was purified from total RNA using poly-T oligo-attached magnetic beads. Fragmentation was carried out using divalent cations under elevated temperature in First Strand Synthesis Reaction Buffer (5×). First strand cDNA was synthesized using random hexamer primers. Lastly, PCR products were purified (AMPure XP system, Beckman Coulter, Beverly, CA, USA) and library quality was assessed on the Qubit2.0 Fluorometer, Agilent Bioanalyzer 2100 system, and using qRT-PCR. The clustering of the index-coded samples was performed on a cBot Cluster Generation System using TruSeq PE Cluster Kit v3-cBot-HS (Illumina, San Diego, CA, USA), in line with the manufacturer’s instructions. After cluster generation, the library preparations were sequenced on an Illumina Novaseq platform (Illumina, San Diego, CA, USA), and 150 bp paired-end reads were generated.

### 2.5. Identification of Differentially-Expressed Genes

The transcriptome raw data from the FASTQ format were first processed by fastp v.0.19.7 [18] with default parameters. Clean reads were obtained by removing reads containing adapters, reads containing N bases, and low-quality reads, from the raw data. After quality control, the Q20, Q30, and GC content of the clean data was calculated. All the downstream analyses were based on the clean data of high quality. Based on OGS (official gene sets) of the previous genome of *E. grisescens* [19], gene-expression levels of all transcriptomes were calculated using RSEM v.1.3.1 [20]. In line with the read- count matrix, DEseq2 [21] was used for expression normalization and differential expression analysis at each time-point. Genes with |log_2_foldchange| >= 1 and padj (adjusted *p*-value) <= 0.05 were recognized as the DEGs. The clusterProfiler v.4.1 [22] was then used for the enrichment of DEGs in the KEGG pathways, using an FDR < 0.05 to filter significant enrichments. Transcriptomic data supporting the conclusions of this article is available at InsectBase (http://v2.insect-genome.com/econpv accessed on 10 October 2022).

### 2.6. Validation of DEGs Using Quantitative RT-PCR

The specific primers used for qPCR were designed using the web tool (https://eurofinsgenomics.eu/en/ecom/tools/qpcr-assay-design/) on 18 November 2021, and are shown in Table 1. Each 20 μL reaction-volume contained 10 μL of ChamQ Universal SYBR qPCR Master Mix (Vazyme, Nangjing, China), 1 μL of each primer (10 μM), 6 μL of sterilized H_2_O, and 2 μL of cDNA template. The qPCR cycling was performed on a Roche LightCycler480 II PCR System (Roche, USA), and the parameter setting was 95 °C for 30 s, followed by 40 cycles of 95 °C for 10 s, 55 °C for 30 s and 72 °C for 20 s. Fluorescence was further measured using a 55~95 °C melting curve to detect a single gene-specific peak. Each reaction was performed with three technical replicates and three biological replicates. *GAPDH* was selected as the housekeeping gene, to normalize gene expression and to correct for sample-to-sample variation, using the 2^−ΔΔCT^ method. The Roche 480 analysis software and the Q-gene method in the Microsoft Excel-based software Visual Basic (Microsoft Corporation, Redmond, WA, USA) were used to calculate the mean normalized-expression levels of the target genes.

## 3. Results

### 3.1. Virulence Determination of the Two EcobNPV Strains

**Figure 1:**
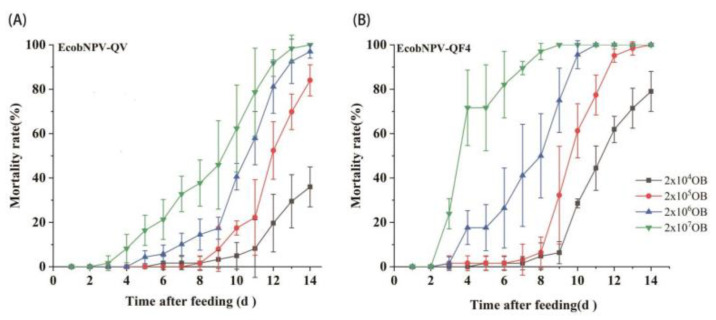
Death dynamics of *E. grisescens* feeding on leaves treated with EcobNPV-QV (**A**) and EcobNPV-QF4 (**B**). Twenty insects in each assay were tested per dose, with four replicates. Larvae feeding on distilled-water-treated leaves were used as control, to calculate the adjusted mortality-rate.

To compare the virulence of EcobNPV-QV and EcobNPV-QF4 strains on *E. grisescens*, we evaluated the virulence of EcobNPV-QV and EcobNPV-QF4 with the leaf-disc method [5]. The cumulative mortality-rates for both strains significantly increased with the feeding time and dosage (Figure 1). The EcobNPV-QF4 strain showed a higher mortality-rate than EcobNPV-QV at the same dose at the same time-point. The mortality was positively correlated with the dose for both groups (correlation rates: 0.97–0.99) (Table 2). The median lethal-dose (LD_50_) of EcobNPV-QV was 55-fold higher than that of EcobNPV-QF4 (4.35 × 10^8^ vs. 7.89 × 10^6^), indicating that EcobNPV-QF4 has higher virulence for *E. grisescens* than EcobNPV-QV.

### 3.2. Transcriptome Sequencing and DEGs Identification

EcobNPV-QV virus particles were reported to vary greatly in copy numbers 48 h after infection [23]. To elucidate the difference of virulence mechanisms between the two virus strains, we sampled larvae feeding on the virus-infected leaf at seven time-points (0, 2, 6, 12, 24, 36 and 48 hpi) for Illumina transcriptome sequencing, yielding a total of 1,395,080,634 raw reads of 410.72 Gb (Table S1). After removing low-quality reads, 1,369,021,419 clean reads were obtained. The base error rate was 0.03%. Q20 ranged from 96.7% to 97.8% (97.2% in average), while Q30 ranged from 91.2% to 93.9% (92.4% on average). The GC content ranged from 46.6~56.1% (49.7% on average). The results suggest that the sequencing data are of high quality, and can be used for further analysis.

**Figure 2:**
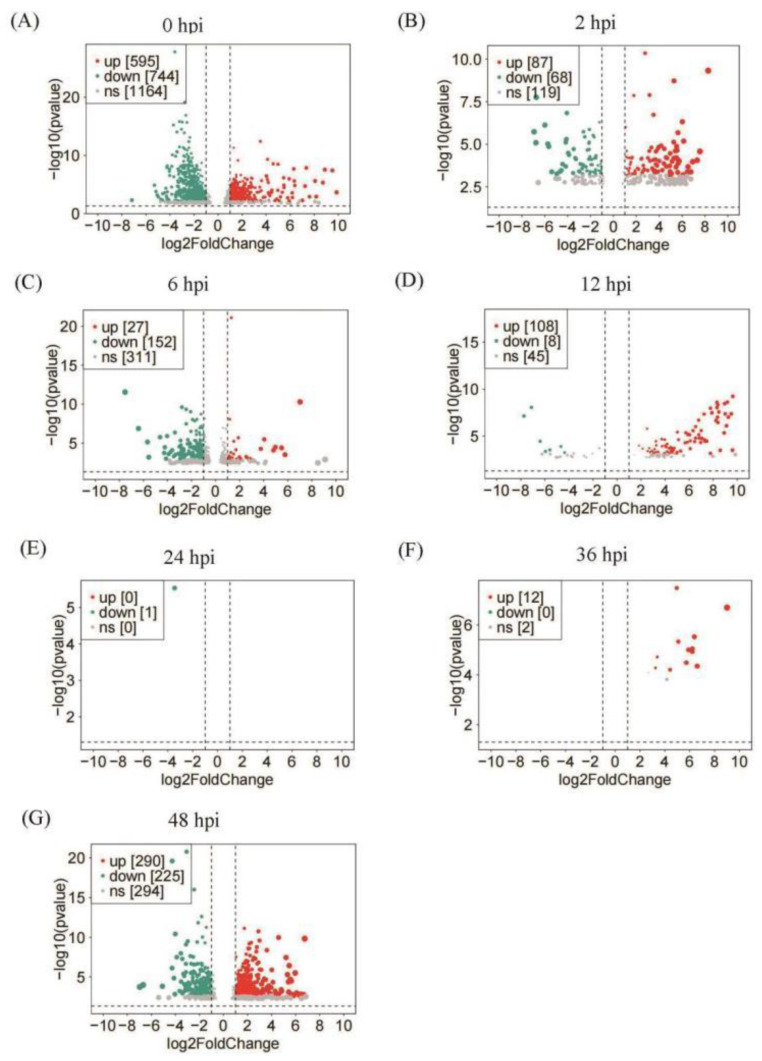
General analyses of host larval expressed-genes responding to EcobNPV-QV vs. EcobNPV-QF4 infection. Genes with |log_2_foldchange| >= 1 and padj (corrected *p*-value) <= 0.05 were recognized as the differentially expressed genes (DEGs). Figures (**A**–**G**) represent the volcano plots of DEGs at 0–48 hpi. Red plots are the genes with significant up-regulated differential expression (up), green plots are those with significant down-regulated differential expression (down), and gray plots are the genes with no significant difference in expression (ns).

In order to identify DEGs between the two virus strains, DESeq2 was used to compare the gene expression at each time-point. The EcobNPV-QF4 infection resulted in a total of 2317 DEGs, including 1119 up-regulated genes and 1198 down-regulated genes (Figure 2 and Tables S1–S3). Among them, 1339, 155, 179, 116, 1, 12 and 515 genes were detected as differentially expressed between the two groups, at 0, 2, 6, 12, 24, 36 and 48 hpi, respectively (Figure 2). Compared with EcobNPV-QV, the EcobNPV-QF4 infection resulted in a high number of 1339 DEGs (744 down-regulated and 595 up-regulated), suggesting that the initial viral invasion caused extensive changes in gene expression. In contrast, only one gene was down-regulated, and 12 genes were up-regulated, at 24 and 36 hpi, respectively. The highest number of DEGs were detected at 0–12 hpi, while only a few genes were differently expressed at 24 and 36 h, implying that the initial infection (within 12 h) is crucial for the discrepancy of gene expression after the larvae are exposed to the two virus strains (Figure 2E–G). The intersection of the top 20 upregulated genes at different time-points showed that *larval cuticle protein A3A* (Gene ID: Eg_07_316_0) was highly expressed at 0 hpi and 2 hpi, and *lipase member H-A* (Gene ID: Eg_05_228_0) was highly expressed at 36 hpi and 48 hpi (Table S4). 

**Figure 3:**
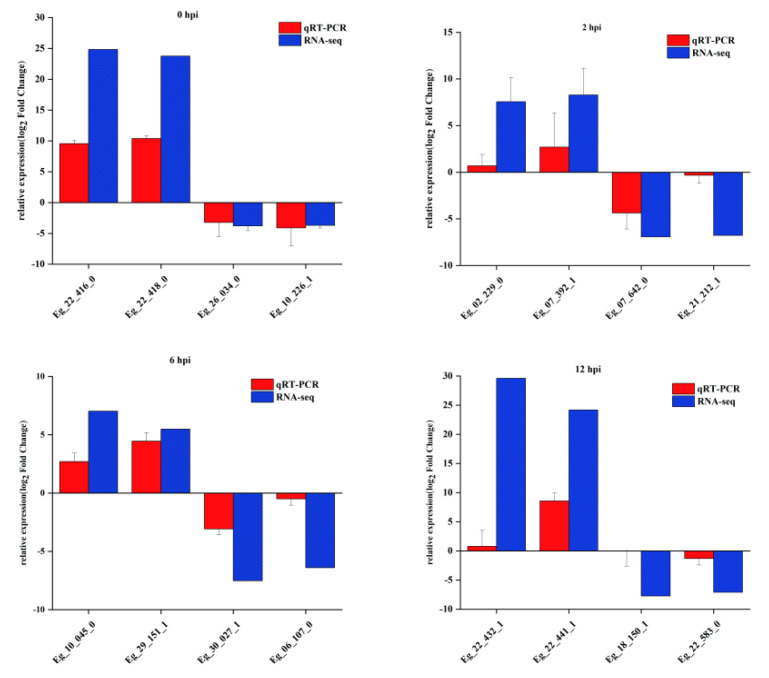
Validation of the differentially expressed genes between larvae feeding on EcobNPV-QV- and EcobNPV-QF4-infected leaf using qRT-PCR. The GAPDH gene was used as a control, to normalize the gene expression of EcobNPV-QV vs EcobNPV-QF4 with qRT-PCR. All data represent mean ± SEM (*n* = 3).

To further validate the reliability of the DEG analysis, we selected two of each of the most-highly up-regulated and down-regulated DEGs at 0, 2, 6 and 12 hpi, with qRT-PCR. Within all the comparisons, the fold changes of the DEGs calculated with the two methods were highly consistent (Figure 3 and Table S5), suggesting the high reliability of the RNA-Seq analysis. For example, *cuticle protein 19* (Gene ID: Eg_22_416_0,Eg_22_418_0, Eg_22_432_1, Eg_22_441_1) were up-regulated and detected with RNA-seq and qRT-PCR at 0 hpi and 12 hpi. *Eukaryotic translation initiation factor 4E* (Gene ID: Eg_22_583_0) was down-regulated 7-fold in the RNA-seq and 1-fold in the qRT-PCR analysis at 0 hpi.

### 3.3. KEGG Enrichment-Analysis

**Figure 4:**
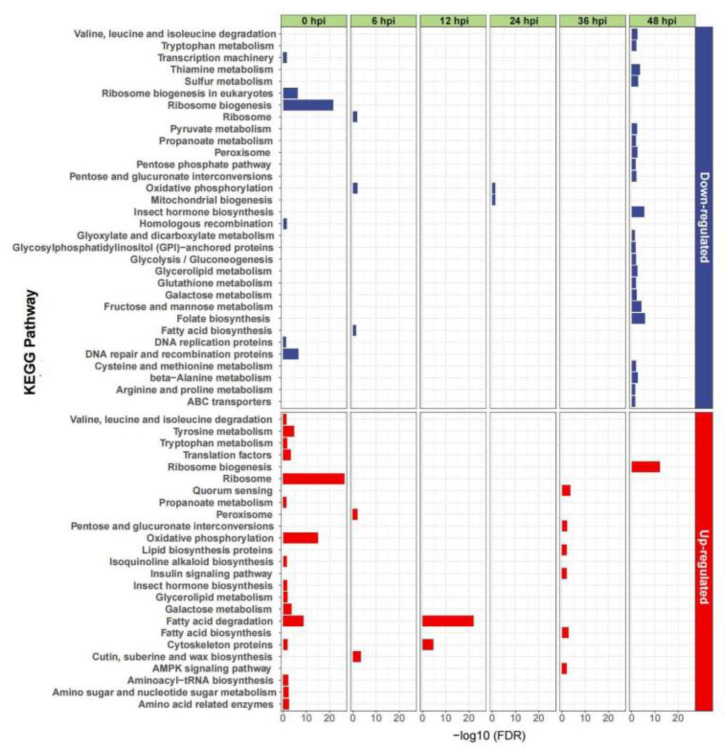
KEGG enrichment-analyses of DEGs among different infected time-points (EcobNPV-QF4 vs EcobNPV-QV). KEGG enrichment-analyses of differentially expressed *E. grisescens* transcripts were first separated into down-regulated unigenes (top portions) and up-regulated unigenes (bottom portions), and then illustrated in EcobNPV-QF4 vs EcobNPV-QV comparison groups in different time (*p*-value < 0.05).

To better understand the gene functions of the host larval-DEGs responding to the virus, KEGG enrichment-analysis was carried out. Among the multiple pathways involved in the EcobNPV infection of cells, we focused on these pathways of up- or down-regulated genes associated with the time after the EcobNPV-QF4 infection. Compared with EcobNPV-QV, genes up-regulated by EcobNPV-QF4 at 0 hpi were significantly enriched in sixteen pathways, including ‘ribosome’ (*p*-value = 2.47 × 10^−29^), ‘oxidative phosphorylation’ (*p*-value = 1.43 × 10^−17^), and ‘fatty acid degradation’ (*p*-value = 3.78 × 10^−11^). As few DEGs were identified at 2, 6, 12, 24 and 36 hpi, this indicates that the host responses to the two virus strains were similar during infection. At 12 hpi, the up-regulated genes in the EcobNPV-QF4-infected larvae were enriched in the KEGG pathways ‘fatty acid degradation’ (*p*-value = 2.15 × 10^−24^) and ‘cytochrome’ (*p*-value = 7.45 × 10^−7^). Meanwhile, at 48 hpi, down-regulated genes in the larvae after the infection of EcobNPV-QF4 were involved in multiple amino-acid metabolism pathways, such as folate biosynthesis (*p*-value = 1.82 × 10^−6^), fructose and mannose metabolism (*p*-value = 8.41 × 10^−5^), and thiamine metabolism (*p*-value = 2.55 × 10^−4^). In addition, only the ribosome-biogenesis pathway was enriched by up-regulated genes, which may account for most of the difference in virulence between the two viruses, as this time-point coincides with the stronger virulence of EcobNPV-QF4 (Figure 4).

**Figure 5:**
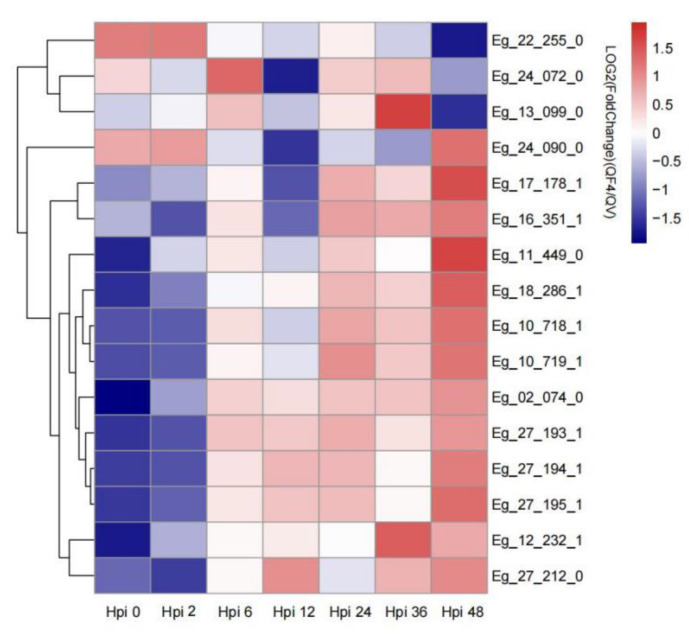
Expression of immune-related genes in *E. grisescens* after ingestion with EcobNPV-QV and EcobNPV-QF4. Each square represents each gene, and the color from red to blue indicates the normalized gene-expression with FPKM from large to small. Hpi: hour post ingestion.

The Toll and Imd signaling pathway, the JAK-STAT signaling pathway and the Toll-like receptor signaling pathway are four pathways known to play a significant role in insect immune-responses [24]. To check whether EcobNPV strains induce an immune response in *E. grisescens*, we identified a total of 16 immune-related DEGs in these pathways (Figure 5). Compared with EcobNPV-QV, immune-related gene *modular serine protease* (Gene ID: Eg_24_090_0), *peptidoglycan recognition protein 4* (Gene ID: Eg_16_351_1), *suppressor of cytokine signaling 2* (Gene ID: Eg_17_178_1) and *CWF19-like protein 2 homologs* (Gene ID: Eg_18_286_1) were up-regulated after EcobNPV-QF4 infection in *E. grisescens*. In addition, thirteen genes involved in immune signaling-pathways were also identified in the DEGs, including those encoding *caspase-8* (Gene ID: Eg_10_719_1), *modular serine protease* (Gene ID: Eg_24_090_0), and *phosphatidylinositol 3-kinase regulatory subunit alpha* (Gene ID: Eg_12_232_1), which were found to be down-regulated compared with EcobNPV-QV (Table 3). Different immune genes may play inconsistent roles in response to two virus infections.

## 4. Discussion

Previously, the genomes of the two virus strains revealed that sequences and palindromes in the homologous repeat-regions might contribute to the discrepancy in virulence of the two strains [10]; however, there is still limited understanding underlying the virulence mechanism. In this study, we confirmed the difference in virulence of the two strains against *E. grisescens*, and conducted comparative transcriptome analysis of the *E. grisescens* larvae infected by the two strains. These results showed that, relative to EcobNPV-QV, the number of DEGs down-regulated by EcobNPV-QF4 was similar to the number of upregulated genes. 

A variety of viral potentiators can bind to specific sites on the periplasm or degrade periplasmic proteins and chitin, thereby disrupting the structure of the periplasm and facilitating the virus infection of insects [25]. The up-regulated genes in the cuticle-, suberin- and wax-biosynthesis pathway at 2 hpi might be induced by the degradation of the periplasmic membrane (Figure 4). Insect immunity to baculovirus can be divided into the defense against ODV in the digestive tract and the defense against BVs in other tissues such as fat bodies, trachea and blood cells. *E. grisescens* feeds on virus-bearing leaves, and the polyhedrovirus is cleaved into virus particles. Occluded ODVs are released from OBs within 12 min post entry into the insect midgut. At 2 hpi virus particles enter the midgut and fuse with microvilli of midgut columnar epithelial cells [26]. In the midgut, ODVs shed their nucleocapsids and replicate in the nucleus, forming BV and ODV-type virus particles [27]. The insect midgut is an important site for the secretion of digestive enzymes, digestion of food and absorption of nutrients, as well as an important target for virus invasion [28]. At 24 hpi, viral particles emerge from the infected midgut, and extend the infection to the whole body. At 36 hpi, viral particles cross the basement membrane, expand via the trachea, and infect the blood body-cavity and other tissues [26].

Virus infection of cells induces autophagy and apoptosis, which play a very important role in antiviral immunity [29,30]. One down-regulated DEG, *phosphatidylinositol 3-kinase regulatory subunit alpha*, is associated with autophagy, and can inhibit cellular autophagy and reduce virus production in larvae bodies (Table 3). Another DEG, *caspase-8*, is involved in the induction of apoptosis, which can inhibit virus replication and promote the clearance of infected cells by phagocytes, thereby reducing or stopping the spread of the virus in the host [31,32]. These genes were expressed at lower levels in *E. grisescens* infected with EcobNPV-QF4, compared with EcobNPV-QV, suggesting an attenuated immune-response against EcobNPV-QF4.

Lipase can reduce infection by the virus in silkworm, leading to the reduction of virus replication, but overexpression disturbs the physiological balance of the host, accelerating the expression of the virus in the body [33]. In *B. mori*, *lipase member H-A* (*BmLHA*) showed up-regulation in the midgut digestive-juice of the resistant strain (A35), compared with the susceptible strain (P50) [34]. A lipase-like gene, (ORF19), from *Heliothis virescens ascovirus* (HVAV-3E) was critical for viral replication and cell division [35]. In the EcobNPV-QF4-infested larvae, *larval cuticle protein A3A* (Gene ID: Eg_07_316_0, at 0 hpi and 12 hpi), and *lipase Member H-A* (Gene ID: Eg_05_228_0, at 36 hpi and 48 hpi) were up-regulated by 44, 495, 21, and 40-fold, respectively, implying that transcriptome responses to the two virus strains might be involved in host immunity.

When the virus first invades the larvae after ingesting the EcobNPV-QF4-treated leaf-discs, pathways such as ‘ribosome’, ‘oxidative phosphorylation’, and ‘fatty acid degradation’ were significantly enriched (Figure 4). The viral infection induces damage to the cell organelles, causing the degradation of fatty acids resulting in oxidative stress [36]. Cytochrome P450s are unique heme-containing proteins, which are essential for the organism’s defense against toxic substances [37,38]. At 48 hpi, down-regulated genes caused by EcobNPV-QF4 were enriched for the amino-acid-synthesis pathways, while up-regulated genes were only enriched for the ribosome-biogenesis pathways, which may be responsible for the stronger virulence of EcobNPV-QF4.

## 5. Conclusions

In this study, we examined the transcriptional responses of *E. grisescens* to the infection of two strains, and compared the pathway changes after infection. The results showed that the immune-related genes of the *E. grisescens* revealed different changes after infection, and sixteen key genes related to immunity were screened. The infection by EcobNPV-QV activates more immune genes, and reduces the activity of the virus. KEGG enrichment showed that the pathway enriched by down-regulated genes was mainly related to amino-acid synthesis, and also affected the biological pathway of ribosome synthesis. This study provides new insight into the baculovirus interactions with the host and provides a theoretical basis for the development of more efficient strains in the future.

## Figures and Tables

**Table 1:** Primer sequences for quantitative qRT-PCR.

Gene ID	Primers (5′–3′)	
Eg_22_416_0	F: CACGATGAATACGCACACC	R: TGCACTACAGCGTTAAATCC
Eg_22_418_0	F: GTTCAGCAAAATCGTAGCTTTC	R: GTATTCATCATGACCGTCGTAG
Eg_26_034_0	F: GAAACCAGAGACGAGGAAAAG	R: TCCGGCGAGGTAATTCAAC
Eg_10_226_1	F: ACAAAAGAGGCAACTGTAACAC	R: GCTTATTCCTGATGCTACCCTG
Eg_07_642_0	F: TTCCGTCTCACAACCCGTTC	R: GCCGCCTAGCAACAAGAAAG
Eg_21_212_1	F: ATTTGAAGCCGCTCGCATC	R: CTTACCAGGAAAGGGTACGC
Eg_02_229_0	F: AAATGTTCATCGTCCTATGCC	R: CAGAAGCAGCAGCATACAG
Eg_07_392_1	F: ACCAACCAAACCACCACAC	R: CCACTAAAGCGGCAAATTCTTC
Eg_30_027_1	F: AGAGCCATCTTCTGACTCC	R: TCTGATACCCACTGCTAACAC
Eg_29_151_1	F: CCGCTTCAACTTCCACTTC	R: CCACCATAGCCTCCATAGTC
Eg_10_045_0	F: ACCAACCAAACCACCACAC	R: CCACTAAAGCGGCAAATTCTTC
Eg_06_107_0	F: CAAATCTTCCCTCACATACCC	R: TGCGCTTTCTTCGATAAATCC
Eg_22_432_1	F: TGTTCTCCAAAGTAGTGTGCC	R: TCGTGTCTCACGATGCTCTG
Eg_22_583_0	F: ACCATCACATCAAACTACCATC	R: ACAACATCAAGCCAAAAACG
Eg_22_441_1	F: TTACTCGTCGCAGCACATC	R: ACACCTTGTACGCGAACTC
Eg_18_150_1	F: TGTCTTCCAATTCAGCAAACTC	R: GCCTCATTCTGTTGTGTTTCC
*GAPDH*	F: TCCCTCAGCGGCTTCCTT	R: AACATCATTCCAGCGTCCACT

**Table 2:** Comparison of the LD_50_, LT_50_ and virulence between the two strains of EcobNPV for *Ectropis grisescens*. *LD_50_: Average doses (OBs) necessary to kill 50% of the larvae population in the test. **LT_50_: Average time (days) necessary to kill 50% of the larvae population in the test. Twenty insects were tested per dose in each assay, and four replicates were performed.

Strain		EcobNPV-QV	EcobNPV-QF4
Number		254	260
Virulence regression equation		y = −1.534 + 0.712x	y = −2.638 + 1.107x
Correlation coefficient R		0.9932	0.9653
*LD_50_ and 95% confidence interval		4.35 × 10^8^	7.89 × 10^6^
		(2.57 × 10^8^–8.16 × 10^8^)	(3.44 × 10^6^–1.40 × 10^7^)
Virulence multiple		1	55.1
**LT_50_	2 × 10^4^ OB	26.42	11.51
	2 × 10^5^ OB	11.89	9.55
	2 × 10^6^ OB	10.13	6.91
	2 × 10^7^ OB	8.15	3.56

**Table 3:** Immune-related gene statistics of DEGs among different time-points after infection. The DEGs were analyzed, and the DEGs related to immunity in three common immune-pathways were screened.

Pathway	Gene ID	Time (hpi)	Annotation	Log_2_FoldChange
Toll and Imd signaling pathway	Eg_02_074_0	0	*Cyclic AMP-dependent transcription factor ATF-*2	−2.293196074
	Eg_10_718_1	0	*Putative uncharacterized protein*	−1.671969275
	Eg_10_719_1	0	*Caspase-8*	−1.326504487
	Eg_24_090_0	0	*Modular serine protease*	2.269082305
	Eg_27_193_1	0	*Putative uncharacterized protein*	−1.814366107
	Eg_27_194_1	0	*Ankyrin-3*	−1.803220912
	Eg_27_195_1	0	*Putative uncharacterized protein*	−1.823257098
	Eg_16_351_1	48	*Peptidoglycan recognition protein 4*	2.732780083
	Eg_22_255_0	48	*Beta-1,3-glucan-binding protein*	−1.929052092
	Eg_24_072_0	48	*F-box/WD repeat-containing protein 1A*	−1.280895798
	Eg_27_212_0	0	*Ankyrin-3*	−1.207470231
JAK-STAT signaling pathway	Eg_17_178_1	48	*Suppressor of cytokine signaling* 2	1.126190772
	Eg_13_099_0	48	*Tyrosine-protein phosphatase non-receptor type 11*	−1.128339941
	Eg_12_232_1	0	*Phosphatidylinositol 3-kinase regulatory subunit alpha*	−2.135074923
	Eg_11_449_0	0	*Suppressor of cytokine signaling 6*	−1.314295799
Toll-like receptor signaling pathway	Eg_12_232_1	0	*Phosphatidylinositol 3-kinase regulatory subunit alpha*	−2.135074923
	Eg_10_718_1	0	*Putative uncharacterized protein*	−1.671969275
	Eg_10_719_1	0	*Caspase-8*	−1.326504487
	Eg_18_286_1	48	*CWF19-like protein 2 homolog*	2.143904653

## Data Availability

The data presented in this study are available in this article.

## Supplementary Materials

The following supporting information can be downloaded at: https://www.mdpi.com/article/10.3390/insects13121088/s1. All the data supporting the results are included in the manuscript and the supplementary files. Table S1: Summary of Illumina RNA-seq reads generated from the two EcobNPV strain infected *Ectropis grisescens*. Table S2: Read counts of *Ectropis grisescens* at various times post infection. Table S3: Read counts of *Ectropis grisescens* at various times post infection. Table S4: EcobNPV-QV infected *Ectropis grisescens* with expressed genes at various times post infection. Table S5. Verification of transcriptome data by qPCR.
